# Mental Health in Prison: Integrating the Perspectives of Prison Staff

**DOI:** 10.3390/ijerph182111254

**Published:** 2021-10-26

**Authors:** Ines Testoni, Irene Nencioni, Maibrit Arbien, Erika Iacona, Francesca Marrella, Vittoria Gorzegno, Cristina Selmi, Francesca Vianello, Alfonso Nava, Adriano Zamperini, Michael Alexander Wieser

**Affiliations:** 1 Department of Philosophy, Sociology, Education and Applied Psychology (FISPPA), University of Padova, 35122 Padova, Italy; ines.testoni@unipd.it (I.T.); irene.nencioni4@gmail.com (I.N.); marbien@wolfsburg.de (M.A.); erika.iacona@unipd.it (E.I.); francescamarrella@outlook.it (F.M.); vittoriagorzegno12@gmail.com (V.G.); francesca.vianello@unipd.it (F.V.); adriano.zamperini@unipd.it (A.Z.); 2 Emili Sagol Creative Arts Therapies Research Center, University of Haifa, Haifa 31905, Israel; 3 Local Office of External Criminal Execution, 35100 Padova, Italy; cristina.selmi@giustizia.it; 4 Local Office of External Criminal Execution, 45100 Rovigo, Italy; 5 Due Palazzi Prison of Padova, Via Due Palazzi, 25/a, 35136 Padova, Italy; felice.nava@icloud.com; 6 Department of Psychology, Alpen-Adria-Universität Klagenfurt, 9020 Klagenfurt, Austria

**Keywords:** prison staff, detainees, healthcare, human rights, qualitative analysis

## Abstract

(1) Background: The Italian Constitutional Court’s decision n. 99/2019 abolished the distinction between physical and psychological health care in the Italian prison system. However, this and other changes to the penitentiary system present challenges to prison staff, which may vary based on their roles and backgrounds; (2) Purpose: To create a process of dialogue and collaboration that include different points of view, needs, and proposals regarding mental health in prisons, this study collects and integrates the perspectives of 91 prison staff who work in various capacities in eight prisons in northeast Italy. (3) Methods: Each participant was involved in either a focus group or a semi-structured interview, and thematic analysis was used to process the resulting transcripts; (3) Results: Through this process, 10 themes were derived that highlight the difficulties of working with prisoners with psychiatric disorders or psychological distress, including lack of human and economic resources, lack of positive communication between prisoners and society and a sense of professional incompetency; (4) Conclusions: Based on these themes, the need for increased points of view, dialogue, and collaboration between prison professionals and between prison and society is discussed, and the current feasibility of treating psychiatric disorders in prison is considered.

## 1. Introduction

### 1.1. Shift towards the Recognition of Mental Healthcare in Italian Penitentiary System

In 2019, the Italian Constitutional Court [1] decided to abolish the distinction between caring for prisoners’ mental and physical health. In the Italian Republic, health is a fundamental right ‘of the individual and in the interest of the community’ that is described and guaranteed by Article 32 of the Constitution [2] (p. 19). Increasingly, the notion of health in the Italian penitentiary system includes both physical and mental health, as encapsulated in the World Health Organisation’s (WHO) definition—‘a state of complete physical, mental and social well-being’ [3]. Starting from these considerations on mental health, intended as a broad concept involving several aspects of life, the following paragraphs will provide an overview of the conditions of the Italian prison system and its population (prisoners and staff) and the effects on their mental health.

### 1.2. Prisoners’ Mental Health

Given that the Constitutional Charter unequivocally affirms citizens’ right to have their health protected, the State must assume responsibility for creating the conditions for health. This is especially true in those contexts where health—and especially mental health—is at risk. Within the penitentiary system, for instance, the issue of health is complicated, as prison is an environment characterised by order, discipline, and control rather than by care and cure [4]. Indeed, prison is mostly considered as a container of deviance, discomfort, pathologies, and addictions, and studies have demonstrated that, inside prisons, mental health is more vulnerable [5,6] and psychiatric pathologies are more prevalent [6,7,8] than in free society. The REDiME study [9] revealed that 58.7% of Italian prisoners currently experience psychiatric disorders, compared to only 8.7% of participants in a comparison group from the general population. Furthermore, a very large epidemiological study (*n* = 15,751) in Italy found that 67.5% of prisoners had at least one disease, and, of these, 41.3% had psychiatric disorders, making this the most common pathology [10].

The most common mental disorders amongst Italian prisoners are psychosis, dissociation, affective disorders, anxiety disorders, personality disorders, substance abuse disorders, and comorbidities [11,12,13,14]. Suicide is much more frequent in prison settings than in the general population [15], and, in Italian prisons, one in every 10 prisoners engages in self-injurious behaviour [16,17]. The onset of mental distress among prisoners is due to various factors, first and foremost of which are environmental conditions: dilapidated facilities, few hours outdoors, insufficient training and work activities, limited personal space due to overcrowding, high temperatures, and so on. Some of these conditions led the European Court of Human Rights to issue a pilot judgement in 2013 condemning Italy for its inhumane prison conditions [18]. However, the problem becomes more complex when we consider that many of those who find themselves in the penitentiary system already suffered from psychophysical conditions before entry. Given that the structural elements of prison favour the emergence of psychiatric pathologies, it stands to reason that prison strengthens existing links between social marginality and psychiatric pathologies.

With these data, the following paragraph will explain how, in the last decades, Italian legislation has attempted to manage the issue of mental health within the prison organisation.

### 1.3. Effects of Italian Prison Legislation on Prison Organisation and Mental Healthcare

In an attempt to comply with the WHO’s European Code for Health in Prisons, which states that (1) prisoners should not be discriminated against in their right to health services; and (2) the restriction of personal freedom can have a harmful impact on the mental health of detainees [19], policymakers have pursued profound changes in the Italian penitentiary system since 2008, through DPCM 1, April 2008 [20]. These changes are intended to better align the penitentiary system and the healthcare system [21], and—as per the Italian Constitutional Court’s decision n. 99/2019 [1]—to ensure that prisoners’ mental health is treated as consistently as their physical health. For instance, the newly adopted community care system extends the public National Health System (NHS) into prisons by means of multidisciplinary Forensic Psychiatry Units (FPUs). Moreover, residencies for the enforcement of security measures (Residenza per l’Esecuzione della Misura di Sicurezza, REMS) have been created to accommodate individuals who are deemed socially dangerous but who cannot be held completely responsible for their crimes due to mental disorders [21,22].

However, since Judicial Psychiatric Hospitals (OPGs) were closed in 2015 [23], offenders with mental illnesses have converged within increasingly overpopulated prisons, transforming the baseline epidemiological situation of prisons and contributing to a highly complex environment [24]. Today, 25% of Italian prisons have an FPU, but the weekly working hours of physicians and psychologists are only 7.4 and 11.7 per 100 prisoners, respectively [25]. Penitentiary police officers (PPOs), who, since the passage of Law 395 in 1990, have had to participate in prisoners’ treatment and rehabilitation programmes, must now aid prisoners with mental illnesses despite lacking the necessary psychological competence [26]. The implementation of dynamic security and open-cell regimes has created additional challenges, especially for PPOs [27]. Finally, the replacement of penitentiary educators with juridical-educational professionals (JEPs) in 2010 created further structural change and role ambiguity [28].

Offenders with mental illnesses have special needs that are not compatible with detention and the prison system [7]. Therefore, the inmate population and prison workers have had to live with various forms of psychological distress in a context that does not guarantee adequate space, training, medical/nursing resources, or psychological personnel to manage the new compliance system.

In light of this complex situation, the Diagnostic, Therapeutic and Assistive Path (PDTA), which provides guidelines for dealing with mentally ill prisoners, has recognised the urgent need to improve the work of multidisciplinary teams [29]. Nonetheless, the cultural and institutional mismatch between psychiatry and justice presents serious obstacles to the development of joint interventions [21,30].

In the next section, we will go into detail about the difficulties which, in consideration of all the data provided so far, prison staff also encounter in carrying out their work.

### 1.4. Prison Staff’s Mental Health and Work-Related Stress

Organisational difficulties, role ambiguity, and environmental conditions are not only putting prisoners’ mental health at risk; they are also affecting the mental health of prison staff [26]. Prison staff are faced with the difficult task of fulfilling afflictive, re-educational, and recovery-oriented functions, as mandated by 1975 reforms, resulting in role ambivalence and distress [22]. In fact, prison seems to be a high-risk work environment with regards to mental health.

Correctional nurses have been found to exhibit high levels of depersonalisation [31]—one facet of burnout—and they suffer from moral distress [32,33]. After conducting focus groups with Italian correctional nurses, Carnevale and colleagues [34] found that structural, organizational, and relational aspects of the work environment were unfavourable, leading to a lack of job satisfaction. For example, the high proportion of mental health concerns amongst prisoners, the need to negotiate boundaries between nursing and correctional activities, and the lack of cooperation between different professionals within the prison all depreciated nurses’ work satisfaction. PPOs, too, are at high risk for work-related stress due to the pervasive, complex, and ambivalent nature of their relationship with prisoners, which places them in close proximity to prisoners’ suffering and desperation, leading to feelings of guilt and powerlessness [35]. In one Italian study, 30% of PPOs exhibited burnout with high levels of both emotional exhaustion and depersonalisation [26].

The literature reports numerous role conflicts for correctional nurses, PPOs and JEPs, also showing how the mental health of the professional is not inseparable from that of the user and vice versa [36,37,38]. Nonetheless, few Italian studies to date have focused on multidisciplinary collaboration or attempted to integrate different prison staff’s perspectives on mental health. Moreover, no Italian studies to date have assessed the perspective of prison directors, heads of PPOs, or heads of FPUs.

## 2. The Research

### 2.1. Aims of the Study

The WHO Guide for Prison Health asserts that ‘Prison administrations have a responsibility to ensure that prisoners receive proper health care and that prison conditions promote the well-being of both prisoners and prison staff’ [19].

In light of the far-reaching reorganisation of the mental health system in Italy’s penitentiary system, the concerning incidence of poor mental health amongst both prisoners and prison staff, and the reported obstacles to cooperation between different kinds of professionals within prisons, the present study aims to explore patterns of meaning in different prison staff’s perspectives on the topic of mental health and increase the understanding of potential risk factors and strategies for improving prisoners’ and prison staff’s mental health.

In so doing, we aimed at creating a narrative, to foster collaboration and mutual support amongst different professional groups within prisons.

We also wanted to understand if a process of dialogue that would highlight the heterogeneity of points of view, considerations, needs, and resolution strategies could be observed by collecting the narratives on the mental health of professional roles working in prison and with the inmate population.

### 2.2. Methodology 

In this study, an action-research exploratory approach has been applied to promote cooperation. This involved participatory research, wherein prison staff were treated as collaborators and as experts of mental health in prison contexts [39]. It also involved the use of focus groups to promote mutual learning and develop greater critical and reflective awareness [40,41,42,43]. We used active learning and transformative learning strategies to reframe potentially stressful events and construct shared knowledge [44].

**Figure 1:**
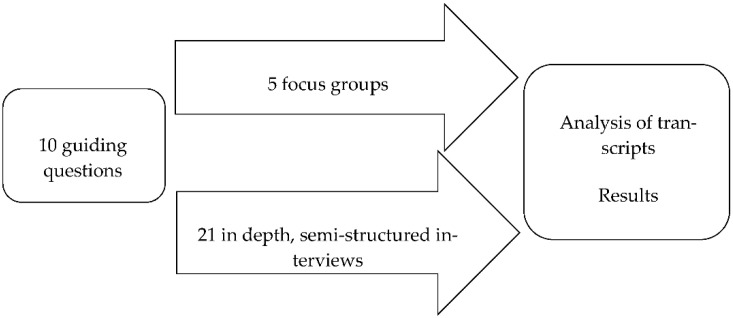
The two parallel phases of the study.

Five focus groups with front-line prison staff (PPOs, educators, doctors, nurses, and a volunteer) were conducted (see Figure 1). Before the focus groups, a presentation to explain the fundamental concepts of action research has been provided; afterwards, the moderator introduced the methodology of the focus group and provided questions to stimulate discussion about the study’s core topics. Following the focus group discussion, the participants were debriefed and invited to share the most significant issues that emerged.

Furthermore, 21 individual interviews with management-level staff (prison directors, heads of PPOs, and heads of FPUs), or so-called ‘privileged witnesses’ were conducted. Each of the twenty-one in-depth, semi-structured interviews lasted about an hour. A flexible approach to these interviews was adopted in order to allow the interlocutors’ needs to direct the course of the conversation. As such, the interlocutors were free to interact and communicate freely, resulting in more open-ended responses [45]. The need to carry out interviews separate from the focus groups with these participants was due to two different needs: (a) to consider whether their representations were very different from those of the operators and therefore their opinions were to some extent on another level; (b) to avoid that their presence in the focus groups could influence the operators or interfere with their perception of freedom of expression.

Each of these engagement modes involved the use of semi-structured questions to promote open dialogue on the following issues: the difference between health and distress; the difference between psychiatric disorders and psychological distress; management of psychiatric disorders and psychological distress after decision n. 99/2019; the materials, strategies, and structural and human resources available (vs. needed) in prison institutions for mental health interventions; work security, and critical incidents that put both workers’ and prisoners’ health and psychological well-being at risk.

To conclude, these issues were discussed with two different samples and two different strategies: for the management-level staff, we adopted the interviews because, due to their role, they possess information that is better explained through an interview.

We preferred to employ the focus-group methodology for front-line prison staff because, according to this approach, each person has his or her own experience and expertise, which, once activated and made available to the community of reference, allows to find a solution. In addition, we wanted to ensure greater freedom of expression to professionals who participated in the focus groups: in the presence of their leaders, the risk was that they would expose themselves to a lesser extent.

### 2.3. Participants

This research is part of a free training project promoted by the Veneto Region, in collaboration with the University of Padua, the School of Public Health Foundation, the Penitentiary Administration Board, and the Juvenile Justice Centre.

Prison staff were informed of this project and the possibility of participating in it voluntarily. The Penitentiary Administration collected the applications and provided us with the participant details.

Participants were 91 prison staff members from eight prisons in northeast Italy (Triveneto region). One of these prisons was a juvenile correctional facility.

Based on their professions, participants took part either in a focus group or in an interview as a so-called ‘privileged witness’. In total, 73 participants with different professional backgrounds participated in the five focus groups. All of these participants worked in ‘frontline’ roles: physicians (*n* = 11), nurses (*n* = 19), JEPs (*n* = 12), psychologists (*n* = 2), volunteer (*n* = 1) (Volunteers within the prison carry out different activities (cooking, cleaning, provision of clothes, etc.), in agreement with the prison staff. They know the rules, the system, they are trained people, so we considered them part of the staff.) and PPOs (*n* = 28). Female staff comprised 49.3% (*n* = 36) of the focus group participants. It was not possible to find information regarding the age or the number of years of service of the participants in the focus groups. 21 participants were identified as privileged witnesses: prison directors (*n* = 7), heads of PPOs (*n* = 6), heads of FPUs (*n* = 6), heads of education units (*n* = 1) and guarantors (*n* = 1). Five of the seven prison directors had previously worked as prison directors at other correctional facilities; four had a degree in law and one had a degree in sociology. With regard to directors and heads of PPOs, it was not possible to find information regarding their age or years of service. As for the heads of FPUs, the average age was about 57, the number of years of service unknown.

All participants signed informed consent forms and were informed of the study’s main results following our analysis through an open conference. The study was conducted according to the guidelines of the Declaration of Helsinki and approved by the Ethics Committee of Università degli Studi di Padova (Protocol Number 3FB6311C0D0C83D3A5B7B28EA1238F9A, 4 February 2020).

### 2.4. Research Team

The research team is composed of five female psychologists, aged between 23 and 26, all with at least two years of experience (research, traineeship, voluntary work) in prisons of Northern Italy; and two scientific heads of the research and supervisors, professors at the University of Padua.

### 2.5. Thematic Analysis

The fundamental aim of the study was to identify patterns of meaning [46] in prison staff’s representations of mental health, as well as to better understand risk factors and strategies for improving prisoners’ and prison staff’s mental health.

Qualitative methods were used to identify these patterns of meaning, as qualitative methods allow researchers to access the most authentic dimensions of participants’ representations [47,48]. This portion of the study proceeded in two parallel phases (as depicted in Figure 1), yielding qualitative transcripts from both the focus groups and the interviews. Because recordings were not allowed in either setting, these transcripts were produced by two independent researchers who compared their work after each data collection session. Both the focus groups and interviews were transcribed and then analysed using the software Atlas.ti, which allows for the development of a theoretical model firmly based on the text [49].

Thereafter, we used thematic analysis [50,51] to parse the qualitative transcripts [52]. This analysis followed the six main phases described by Braun and Clarke [51]: preparatory organisation; generating categories or themes; coding data; testing emerging understanding; searching for alternative explanations, and writing up the report. This involved attributing codes to sections of the text and then deriving semantic networks and main thematic areas. Afterwards, the two—focus groups and interviews—were integrated to create a common understanding from the different perspectives.

Concerning the qualitative validity of the analyses, the research team considered the following aspects. In order to respect the “credibility” (internal validity), two researchers constantly compared the analyses, and a third researcher judged the decisions taken at each step. Furthermore, the results were shared with some participants in order to have feedback along the process.

For the “transferability” (external validity) and the “dependability” (reliability), the involvement of representative participants of each profession permitted researchers to reach all the possible viewpoints.

With respect to the “confirmability”, all the authors of the article were constantly questioned and compared for their different competencies in the field of prison work to consider the meaning of the whole research/action experience and the correspondence with the collected texts. Whenever problematic nodes appeared, meetings were organised via. Zoom because of Covid to solve the objections. Finally, all participants took part in a final meeting via. Zoom to be informed of the results, which received full recognition of validity.

## 3. Results

Ten common themes were derived from thematic analysis of the focus group and interview transcripts:

(1) Definition of distress. Both the prison workers and privileged witnesses defined distress as an alteration of psychological, physical, and social balance. Participants described mental distress as a contextual and transitory condition that is therefore distinguishable from psychiatric pathology, which is permanent. In addition, many defined mental distress as the ‘product of prison’, reporting that the prison environment negatively affects not only prisoners but also the staff. Participants discussed the emotional contamination that mental distress causes within the staff–inmate relationship, which is characterised by mutual influence. A JEP, stated, ‘[when] I begin to sense the weight of the distress, instead of feeling able to help or to compensate the distress, it manifests as a contagion, as if he had thrown his distress onto me’ (1; 85). Alternatively, the participants also struggled to distinguish between a detainee’s genuine discomfort and their performance of distress, which is sometimes used—to the prison staff’s chagrin—as a way to gain certain benefits.

(2) Definition of mental health. All of the participants considered mental health to be an essential part of global health, as defined by the WHO. One prison director expressed it as acknowledging health ‘in 360 degrees’ (1; 6). Another interviewee stated that, ‘here [in prison], one really understands what the WHO’s definition means, what it means to speak of health in global terms, in terms that include all the dimensions of the human being, and how these dimensions are interdependent’ (3; 15).

(3) Definition of psychiatric pathology. Psychiatric pathology was consistently defined as the set of disorders referenced in the DSM-5, and it was strongly associated with the specialised, medical circuit of diagnosis and treatment. Prison workers in the focus groups emphasised the importance of accurate diagnoses, which are often difficult to obtain due to the overlap between genuine pathology and psychological distress. A correctional physician expressed the resulting confusion like so: ‘It is nearly impossible to distinguish between health and distress because health is an equilibrium gained within society, within a community, as we learn from the WHO’s definition. Various factors can compromise mental health [...] it is like a continuum’ (2; 97). Some interview participants reported that psychiatric pathology may precede incarceration or arise as a consequence of it, as an exacerbation of a state of distress due to imprisonment. Participants in both the focus groups and the interviews noted that the entry of an inmate with a psychiatric pathology often destabilises ‘healthy’ prisoners. Three experts also highlighted the tendency to ‘psychiatrise’ behavioural disorders—that is, the tendency amongst staff to overestimate the presence of psychiatric pathologies.

(4) Prison workers. Participants in both phases agreed that staff are expected to work to the best of their ability with available resources, despite the fact that the presiding system does not offer strategies or norms for relating to the patient-inmate. Many also highlighted common states of mind in the workplace, including frustration and a sense of abandonment that can, in extreme cases, reach the point of work-related stress and burnout. Finally, many participants acknowledged the importance of synergistic collaboration between all professionals working in the prison.

Focus group participants claimed that they often perform duties that are beyond the scope of their employment contracts, requiring them to use their own personal, rather than professional, resources. Additionally, they explained that they try to maintain a protective distance from the prisoners to avoid getting too involved in their discomfort. One PPO stated that:

We shouldn’t be subjected to the prisoners’ problems, it is not normal. […] I have adopted the technique of not listening to anyone. There are colleagues who listen to the prisoners’ problems; [the prisoners] throw their problems on them and the colleagues absorb them. But what kind of help can I give? I can only help until a certain point. I can’t provide psychological help; this is why I send them to talk to someone, even though there are very few trained staff members (1; 64).

During the interviews, privileged witnesses described various recreational interventions that have been implemented to protect the well-being of organisational and prison staff, such as yoga lessons and football matches. Privileged witnesses also highlighted the fact that prison staff, and especially PPOs, are reluctant to seek professional psychological support because they feel judged.

(5) Critical issues inside the prison. All participants reported that the crumbling state of prison infrastructure is not suitable for receiving and treating psychiatric patients. Furthermore, all participants indicated a shortage of personnel, and many acknowledged a lack of sufficient training or of dialogue between staff in different professional roles and departments. Prison workers affirmed that they do not have enough tools to manage prisoners with psychiatric problems, and participants in both phases reported increasing numbers of prisoners and the overpopulation of Italian prisons. All participants ntioned the rising number of foreign prisoners, prisoners with addictions, prisoners with behavioural disorders, and prisoners with psychiatric illnesses.

This theme also includes descriptions of factors that can disturb the psychophysical balance of prisoners and prison staff, such as adverse living conditions inside the prison; negative relationships between prisoners; manipulation of psychologically unwell prisoners by healthy prisoners; exacerbation of personal distress due to the prison environment; and excess open-ended time. The head of nurses at one prison reported, ‘As for my experience, the problem is that they [the prisoners] have nothing to do. We organise courses, but after the third day, they don’t attend anymore. One should […] find them something to do—work, school…’ (2; 35). However, while the participants in the focus groups acknowledged these concerns, none of the interview participants reported specific critical events that undermine the mental health of prisoners and prison staff.

Finally, both front-line and management-level staff stressed the need for better internal communication and procedures, reduced role ambiguity, and psychological support. For instance, a JEP stated:

The organisational system lacks the capacity [to address these problems]: we belong to three different areas, and, except for emergencies, there is no real dialogue, there is no designated team for prisoners with particular problems, there is no moment of confrontation; it occurs only from time to time (1; 69).

(6) Critical issues outside the prison. Prison workers reported feelings of dissatisfaction due to the perceived indifference of upper-level penitentiary staff and of the government. Additionally, they complained about the lack of communication between prisoners and the outside community, which should play a key role in reintegrating prisoners into society and preventing recidivism. A PPO in a juvenile correctional facility said:

The outside community should intervene in order to prevent [recidivism], otherwise, the re-educational work we are doing inside of the prison will be in vain. […] in Italy, one or two of every 10 ex-offenders return to prison. If, once they find themselves outside of prison, they encounter a void or an absence of support, they [are likely to] return to committing crimes. It is then that we see if [re-education] is working, if the recidivism rate is decreasing. And it is not the prison’s fault, but the system’s (3; 100).

In the interviews, privileged witnesses focused primarily on the lack of economic resources to renovate prison buildings and on the chronic lack of staff. It emerged that health professionals work in prisons for only a few hours per week and that this affects the continuity of treatment for various pathologies, both physical and mental. Instead, healthcare tasks are often delegated to the PPOs, who are forced to manage situations that are beyond their competence.

(7) Resources. This theme addresses the resources necessary to cope with the above-mentioned issues. Participants in both phases of the research listed the following key existing resources: multidisciplinary team meetings; collaborative work and cooperation in both emergency and ‘normal’ situations; and satisfactory and fruitful relationships between colleagues. During the focus groups, participants pointed out that small prisons are more open to the outside community than larger prisons, and that this is a valuable resource because it promotes continuous commitment to new treatment activities for prisoners. Furthermore, focus group participants reported that it is easier to access care in small prisons, where the number of prisoners is low, than in larger prisons. Privileged witnesses discussed the central role that recreational, work, and treatment activities play in fostering a better prison climate.

(8) Strategies for coping with psychological distress. This theme assembles the actions and strategies that are implemented in individual prisons to address and prevent mental distress. In both phases, participants credited multidisciplinary interventions and the ability to listen and mitigate a person’s discomfort. During the focus groups, participants focused on the importance of getting to know the prisoners, listening and talking to them, and offering them strategies for re-entering society. Michela, a prison volunteer, stated that:

Listening is as important as knowing the person. When a person feels known, they have more confidence, and you are able to contain them. Observing and collaborating is crucial, and it arises from the goodwill of individuals, not from the institution (2; 20).

For mentally unwell patients, these actions can lower distress and decrease reliance on psychotropic drugs. During the interviews, privileged witnesses focused on strategies like early intervention, behavioural protocols, and educational and recreational activities carried out by cooperatives and volunteers, which help to de-escalate uncomfortable situations.

(9) Treatment of psychiatric pathologies. This theme describes the existing resources available for the treatment of persons with psychiatric pathologies. In both phases, participants favoured the following three interventions: (1) sending the patient to a hospital for mandatory health treatment (Trattamento Sanitario Obbligatorio, TSO) as in most cases psychiatric pathologies cannot be treated adequately in prison; (2) preventatively paying attention to ‘at-risk persons’—i.e., those prisoners whose behaviour could lead to a critical event (such as suicide)—including screening newcomers; (3) contacting the health personnel working within the prison (e.g., psychiatrists, psychologists, and pathological addiction service psychologists).

In the course of discussing existing treatment avenues, several participants referenced departments of their correctional facilities that are dedicated to mentally semi-injured prisoners or to prisoners with pathological conditions that emerged during detention. One privileged witness pointed out that such departments often lack adequate managerial training when they are first created. Another participant working in the same institution pointed out the difficulty of managing the patients in these departments, as they interact with both specialised medical staff and with PPOs, who do not have adequate training to properly manage these prisoners.

(10) Proposals. Participants in both phases were asked to indicate the first improvement they would make to the prison where they work if they had the chance. Common responses in both phases were improving communication between treatment areas; emphasising the strategic importance of the outside community; facilitating each prison’s communication with the outside world; further engaging prisoners in recreational and work activities inside the prison; giving more consideration to alternative sentences completed outside the prison; and increasing training courses for prison workers and PPOs. With regard to the last suggestion, two related potential improvements were (1) encouraging prison guards to spend more time in psychiatric departments, collaborating with local health authorities to see how psychiatric pathologies can be managed on a day-to-day basis, or (2) including specific psychology modules in the training of PPOs in order to ‘improve confrontation techniques and bolster relationships with prisoners who exhibit distress or pathology’ (3; 14)—as stated by a focus group participant. Additionally, some suggested simply not admitting offenders with diagnosed psychiatric pathologies into prison. During the focus groups, participants proposed constant monitoring of suicide risk, increasing the number of REMS (specific residencies for offenders with psychiatric problems) in the external community, and hiring more staff through open competitions. The privileged witnesses underlined the need to increase the number of psychologists in prison, as well as their working hours, as they can serve as a resource for both prisoners and prison staff.

(11) Deflection. One last issue that has not been included in the aforementioned themes relates to the ‘deflection’ of responsibilities between prison administrators, such as PPOs, and health workers when it comes to managing prisoners with psychiatric pathologies and psychological distress. Once again, organisational difficulties between different kinds of prison staff emerged, as reported by the head of an FPU:

Another critical aspect in the management of patients is the conflict with prison administrators like PPOs [...]. They, […] would prefer that the patients be treated as they are in REMS, but we cannot provide this autonomy in their treatment because we are still inside of a prison. The 24-hour management [of patients] is their responsibility (12; 23).

## 4. Discussion

These results clarify different prison staff’s perceptions of mental distress and mental illness. Namely, the participants considered mental health to be an important part of overall health, as defined by the WHO; they considered psychiatric disorders to be those defined by the DSM-5; they considered mental disease to be, to a certain degree, a ‘product of prison’.

As noted in the ‘Prison workers’ and ‘Critical issues inside the prison’ themes, prison workers—and especially PPOs—showed little confidence in their professional skills. Consistent with previous literature, participants believed that they were not sufficiently trained to handle certain situations, and they felt that they had to complete tasks that surpassed their professional competence, consequently relying on personal skills, which is causing significant discomfort [27,35,53,54,55,56]. Also, consistent with previous literature, the privileged witnesses reported that it is difficult to deal with the issue of staff’s mental health and poor professional competence because prison workers, and especially PPOs, are reluctant to seek psychological help [57]. It might be useful to investigate whether this self-perception could become a radicalised belief. At the policy level, interventions should be configured to increase staff’s role identity and their confidence in their own competencies. This is essential, as poor self-confidence can lead prison staff to engage in dehumanising prisoners in order to avoid involving themselves in the prisoners’ distress [58,59,60].

From the ‘Critical issues inside the prison’ theme, we see that prisoners struggle to make use of their time in prison. Based on the principle of non-discrimination, prisons provide vocational, educational, cultural, recreational, and athletic activities for prisoners—and especially minors—to prevent marginalisation and enable social re-reintegration. However, not all prisoners avail themselves of these opportunities, as spaces are not adequate to accommodate everyone. As of 31 December 2019, there were 18,070 (29.74%) prisoners involved in a work activity, even if only for a few hours per week. Very rarely has the percentage exceeded 30% in the last ten years. The vast majority were employed by the prison administration itself (86.82%), mainly in prison services (82.3% of this share) related to cleaning, meal delivery, and other small tasks [25].

Regarding the ‘Critical issues outside the prison’ theme, it is notable that prison workers reported a lack of support from the prison administration itself. It would be useful to look into this issue in order to uncover what causes staff to think this way. Also noteworthy was the fact that all participants acknowledged a lack of communication between the prison and the external community, the latter of which was regarded as a precious resource for the well-being of both prisoners and prison staff.

**Figure 2:**
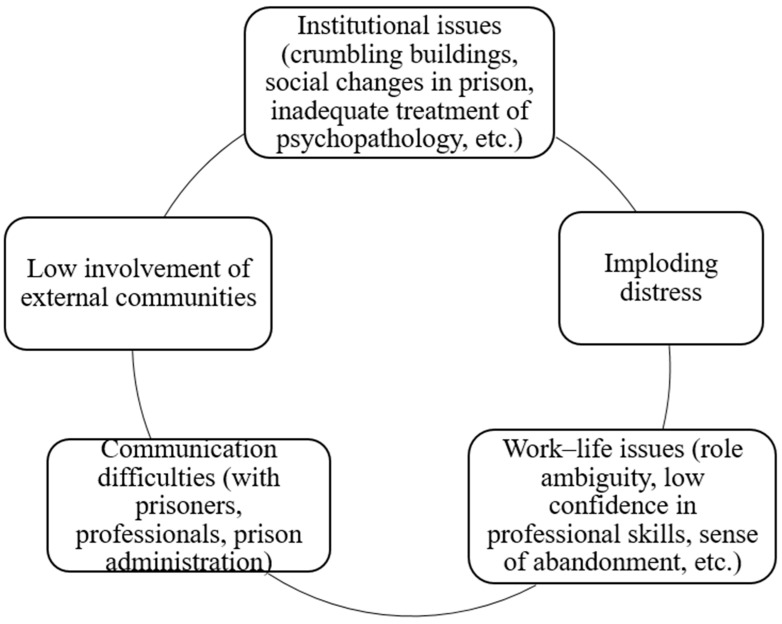
Circle of critical points.

Participants affirmed that they wanted to initiate a process of ‘expansion’, or openness, building relationships of trust between the inside and outside of the prison, in contrast to the process of ‘implosion’ wherein prison activities are entirely contained within the prison walls (see Figure 2 for an illustration). This need should not be underestimated, and future studies should investigate ways to promote dialogue between prisons and their surrounding communities.

However, inflexible bureaucratic systems, unskilled communication, and misinformation about prisons in the surrounding community can complicate these efforts.

In terms of ‘Resources’, participants cited training courses, multidisciplinary work, team spirit, good relationships with colleagues, and recreational activities for workers as key resources for a good organisational climate, as stated in previous literature [61,62]. These resources operate as protective factors against work-related stress, burnout, job dissatisfaction, and psychological distress, and they are, thus, a valuable starting point for future interventions. In this sense, it is essential to increase relationships of trust, communication skills, and understanding of other workers’ needs to generate a common narrative that promotes collaboration between different professional groups within the prison. This will involve encouraging dialogue between different professions; building clear professional role identities; promoting accountability and participation; and implementing strategic communication to promote empathy toward prisoners and their needs.

All participants cited communicating with prisoners, intercepting their discomfort, and empathic listening as ‘Strategies for coping with psychological distress’. Contrary to what is reported in the literature [58,59,60], in this case, the following emerges that the participants do not typically dehumanise prisoners, but rather regard them as people with their own needs: a strategy identified to move towards well-being is empathy towards the prisoners.

Finally, most participants affirmed that serious psychiatric pathology cannot be treated in prison. This corresponds with the Italian Constitutional Court’s stance, as articulated in decision n. 99/2019, that the lack of alternatives to prison for those with serious mental illnesses constitutes a lapse in the protection of one’s right to health, amounting to inhuman treatment that undermines the detainee’s health.

From these results, we can highlight that, although the interviewees have different roles, there is a homogeneous narrative, which does not present strong characterisations: within a total institution, such as the prison, the participants seem to have the same asphyxiated representation of mental distress.

## 5. Limitation of the Study and Future Developments

Since the attendance at the study was voluntary, not all the staff and the privileged witness of the institutes made themselves available to participate in interviews or focus groups. This may be a limitation, as more participation was expected. Despite this, the willing participants were representative of the active and proactive subjects of each Institute.

Another limitation, and future development, is the lack of focus on the relationships between the identified categories. Based on these data, in the future, we can structure a qualitative study with the specific aim of tracing the relationships between them. In addition, we could pay attention to sociodemographic and professional information about participants to hypothesise correlations with the categories that are identified by this research.

One of this work’s main limitations is that it is “action research”, i.e., it aims to investigate an aspect of social reality by activating consciousness-raising processes. This inevitably implies that the reality within which the research is carried out changes as the researchers work with the participants, promoting their awareness of the problem they are reflecting on together. Furthermore, the simultaneous involvement of different professions may have created a mutual influence, limiting the possibility of getting to know each profession’s specific points of view.

Moreover, since all researchers were involved in the research process, unified by the idea that action-research can be an effective method for the consciousness-raising process, the discussion of the data collected is likely affected by the starting point of the whole working group.

On the other hand, these same limitations can be considered a strength insofar as they can initiate virtuous integration processes between social research and intervention to improve the quality of human relations.

Considering this project as a first local experiment, in the future, we would like to expand this kind of survey to more Italian regions.

## 6. Conclusions

In the last few years, there have been crucial and far-reaching changes to the strategies used to deal with mental health in Italian prisons. The present research aimed to shed light on both sides of this issue by exploring the interrelation of prisoners’ mental health and prison staff’s mental health and by assessing and integrating the perspectives of prison staff in different organisational roles, from frontline workers to prison directors and PPOs to healthcare staff. The results illustrate a highly complex transformative process in prison institutions and the ongoing struggles faced by various prison staff. In particular, the ‘Deflection’ theme highlights the discordant cultural and organisational structures at play and the varying conceptualisations of responsibility held by different kinds of staff. These present significant obstacles to effective collaboration across prison departments. The participants in this study expressed the urgent need to pursue new modes of collaboration, both inside the prison (e.g., by increasing team meetings, protocols, and training) and with the outside community. Many participants in both frontline and management positions criticised the presence and treatment of prisoners with mental illness. Indeed, the institutional conflict between containing and curing psychiatric illness also causes psychological distress amongst prison staff. Even though Italian prison legislation focuses on re-educating prisoners, prison staff also require additional resources, training, and support to mobilise internal and external networks and thereby reduce recidivism. According to the participants, these collaborative connections are vital to improving mental health conditions for prisoners and prison staff alike.

## Data Availability

The dataset used and/or analysed during the current study are available from the authors on reasonable request.
